# Electrosurgical hysteroscopic metroplasty for septate uterus: a single-center retrospective analysis of safety, adhesion rates, and perioperative outcomes

**DOI:** 10.1007/s00404-025-08169-2

**Published:** 2025-09-04

**Authors:** Elvin Piriyev, Sven Schiermeier, Thomas Römer

**Affiliations:** 1 University Witten-Herdecke, Witten, Germany; 2 Department of Obstetrics and Gynecology, Academic Hospital Cologne Weyertal, Cologne, Germany; 3 University of Cologne, Cologne, Germany

**Keywords:** Septate uterus, Müllerian anomalies, Hysteroscopic metroplasty, Monopolar, Bipolar

## Abstract

**Objective:**

To evaluate the safety, adhesion rates, and perioperative outcomes of monopolar and bipolar electrosurgical hysteroscopic metroplasty in women with a septate uterus.

**Methods:**

We conducted a single‐center retrospective analysis of 155 consecutive patients who underwent same-session laparoscopy and hysteroscopic septum resection between January 2021 and January 2025. Procedures were performed under surgeon discretion using either a monopolar loop with glycine distension or a bipolar loop with isotonic Ringer’s lactate. Postoperative prophylaxis against intrauterine adhesions comprised hyaluronic acid gel—with or without a copper intrauterine device—and, in selected extensive resections, a three‐month estrogen–progestin regimen. Endometriosis was diagnosed laparoscopically and, when lesions were excised, confirmed histologically.

**Results:**

The most common indications for the surgery were endometriosis (40.6%), recurrent pregnancy loss (38.0%), and infertility (19.3%). Septal morphology was subseptate in 67.7%, septate in 20.0%, and complete septate in 12.2%, and bipolar energy was used in 65.1% of procedures. No uterine perforations, fluid-overload syndromes, or major hemorrhages occurred, and one case of postoperative endometritis (0.6%) was recorded. Second-look hysteroscopy, performed in 69 patients (44.5%), showed intrauterine adhesions in 3/69 (4.3%; grade I 2.9%, grade II 1.4%); residual septal tissue was observed in 50/69 (72.5%), predominantly in extensive septa. Histologically confirmed endometriosis, identified on concomitant laparoscopy, was present in 126/155 (81.3%) and did not differ across septal types (*p* = 0.103).

**Conclusions:**

Monopolar and bipolar electrosurgical hysteroscopic metroplasty showed a good safety profile with negligible major complications, low adhesion rates, and minimal infection. Bipolar systems further enhance safety by enabling isotonic fluid use.

## What does this study add to the clinical work

**Table:** 

Electrosurgical hysteroscopic metroplasty showed a good safety profile in 155 cases, with no perforations, major hemorrhage, or fluid‐overload events and only one endometritis (0.6%). On selective second look, intrauterine adhesions were infrequent and mild (4.3%).	

## Introduction

A septate uterus is the most common congenital Müllerian anomaly, characterized by a fibrous or fibromuscular partition dividing the uterine cavity [1–3]. It occurs in approximately 2–3% of the general female population, with a higher prevalence among women with infertility or recurrent pregnancy loss [4–8]. The presence of a uterine septum is strongly associated with adverse reproductive outcomes, including markedly elevated miscarriage rates (reported as 36–77% in septate uteri versus ~ 10% in normal uteri) and reduced live birth rates (around 38% vs 85% in women without anomalies) [2, 3]. These effects are thought to result from the septum’s impact on intrauterine volume and endometrial function, which can impair implantation and lead to early pregnancy loss [9].

Emerging evidence also suggests a link between septate uterus and endometriosis. A high coincidence of endometriosis has been observed in women with uterine malformations: one series reported endometriosis in ~ 75% of such patients, with rates approaching 93% in women with a complete septate uterus [10]. This association may reflect shared developmental factors or altered menstrual outflow (leading to increased retrograde menstruation), underscoring the importance of evaluating for endometriosis in patients with septate uteri [10]. Both endometriosis and a septate uterus can independently impair fertility, so their concurrence is a significant clinical consideration.

Given the negative impact of a septate uterus on fertility, surgical intervention is often recommended to restore normal uterine anatomy and improve reproductive outcomes [2, 11]. Hysteroscopic septum incision (metroplasty) is the treatment of choice, having largely replaced the older abdominal metroplasty due to its minimally invasive nature and high success rates [2, 11]. Numerous studies have documented improved obstetric outcomes after hysteroscopic septum resection; for example, miscarriage rates reportedly drop from ~ 94% to ~ 10% and live birth rates rise from ~ 2% to > 80% after septum removal [2, 9, 11].

Attention has turned to optimizing hysteroscopic techniques and energy modalities to maximize safety and efficacy. Traditionally, monopolar electrosurgery has been used for septum dissection, which requires a non-conductive distension medium (e.g., glycine). Newer bipolar hysteroscopic systems permit use of isotonic saline, greatly reducing the risk of fluid overload and hyponatremia [12]. Comparative studies indicate that bipolar septum resection achieves similar operative and reproductive outcomes to monopolar resection, while offering a safer fluid management profile [12]. Both methods are generally effective and well tolerated, although careful technique is required to avoid complications such as uterine perforation or intrauterine adhesions.

For extensive septa (e.g., a complete uterine septum extending to the cervix), advanced surgical approaches have been introduced. Römer et al. described a combined transcorporal hysteroscopic septal dissection using a balloon as a guide, which successfully created a normal cavity in most patients and led to favorable fertility outcomes. This technique was deemed safe and effective in treating complex septate uteri [13, 14].

The aim of this study was to evaluate the safety of monopolar and bipolar hysteroscopic septum dissection, with a focus on second-look hysteroscopy findings, intrauterine adhesion formation, and peri- and postoperative complications.

## Materials and methods

This retrospective case series was conducted at Academic Hospital Weyertal. We identified all patients who underwent hysteroscopic septum dissection between January 2021 and January 2025. Only those who received operative treatment were included, yielding a total cohort of 155 cases. Procedures were performed under the surgeon’s discretion using either monopolar or bipolar electrosurgical energy. Monopolar dissection employed an electrolyte-free distension medium (Purisole), whereas bipolar resection utilized isotonic Ringer’s lactate.

**Fig. 1:**
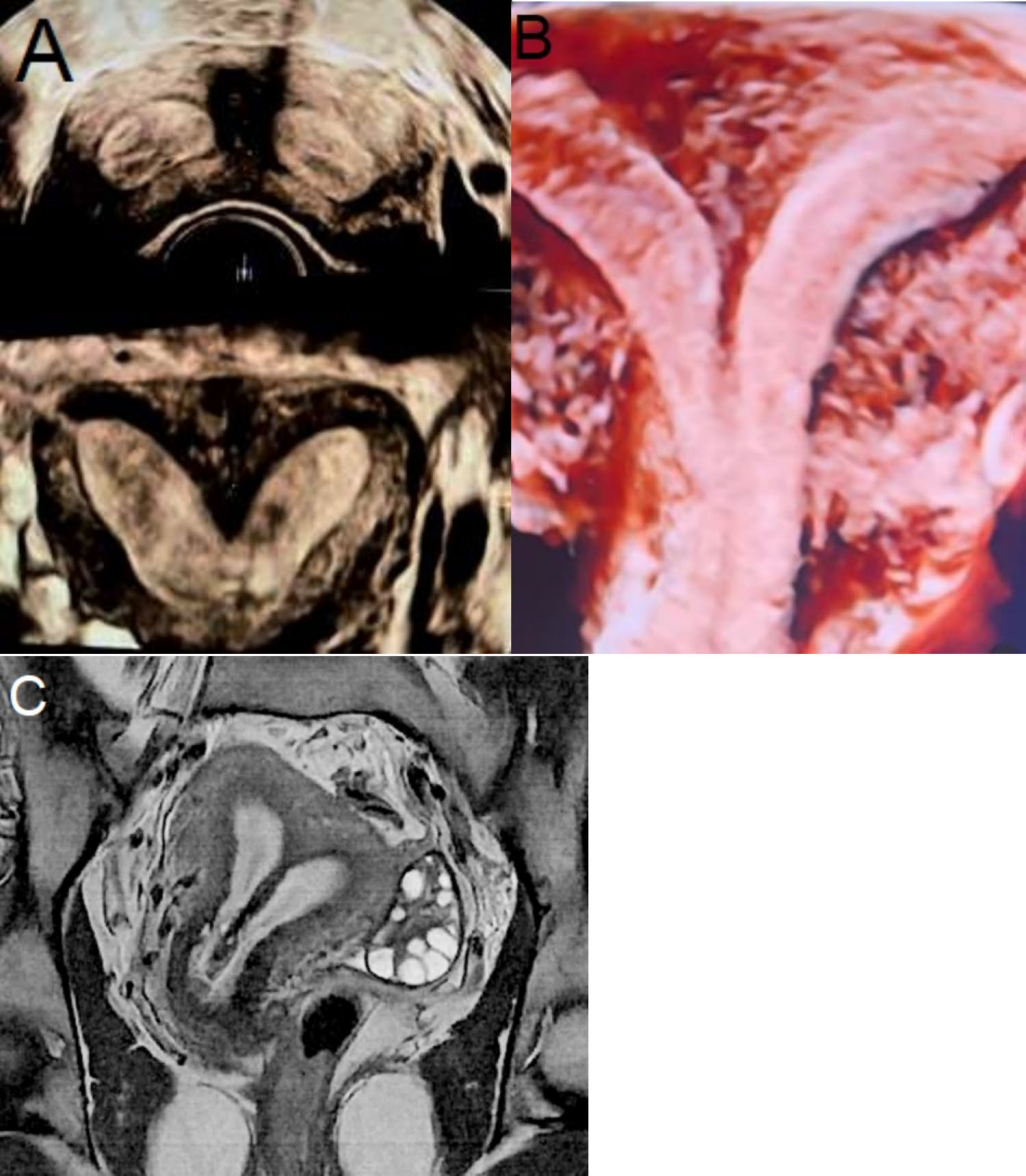
Imaging tools. **A** Transvaginal ultrasound (subseptus), **B** Transvaginal ultrasound (subseptus), C- Magnetic resonance imaging (septus)

**Fig. 2:**
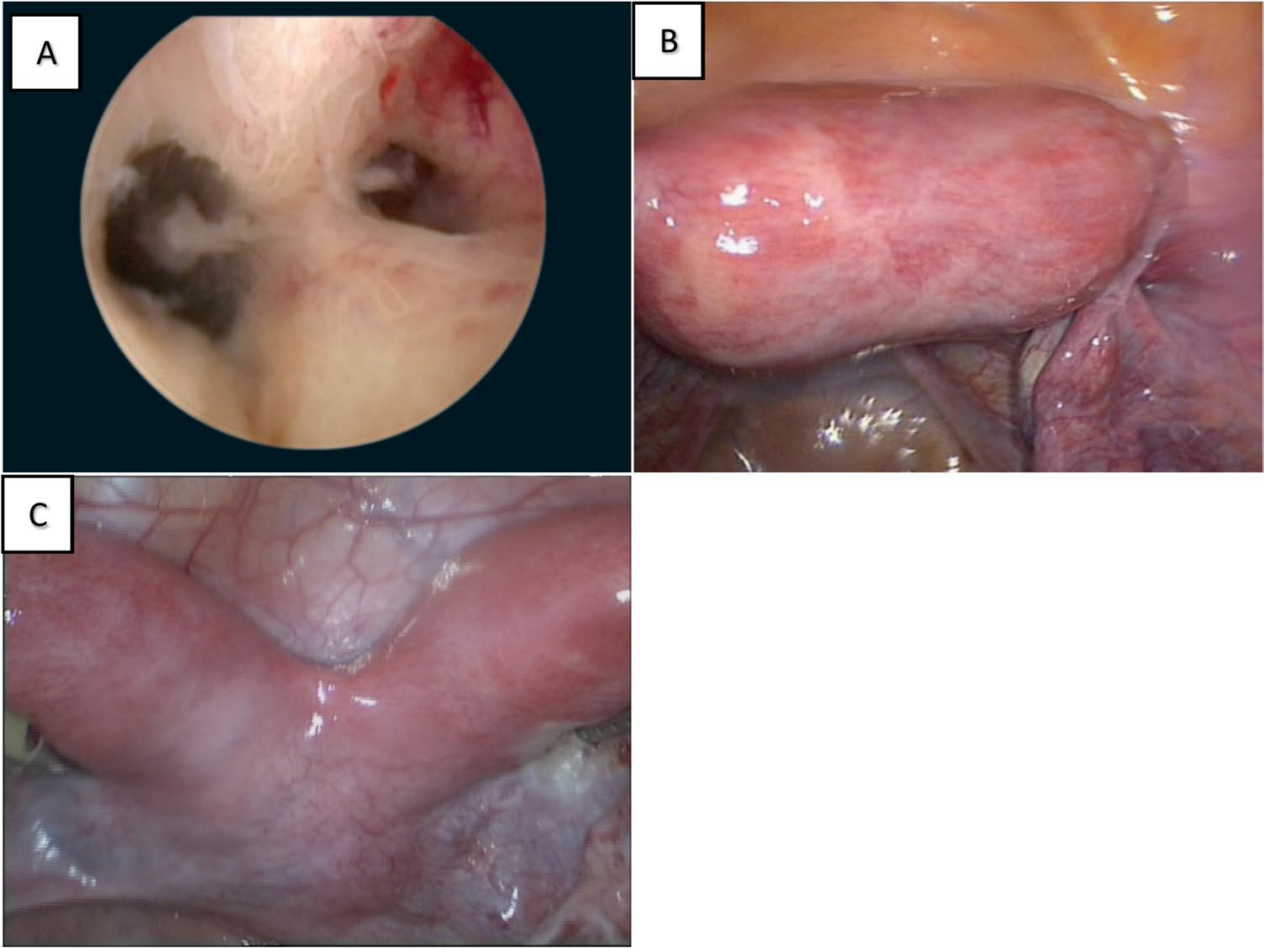
Endoscopic assessment. **A** Hysteroscopic evaluation. **B** Laparoscopic evaluation: conforming septate uterus. **C** Laparoscopic evaluation: bicornuate uterus (differential diagnosis)

Preoperative evaluation consisted of transvaginal ultrasound or magnetic resonance imaging (Fig. 1), supplemented in our center by laparoscopy. Laparoscopy not only permits direct assessment of uterine anatomy but also allows concurrent identification and treatment of endometriotic lesions. Direct visualization of the exterior and interior of the uterus using laparoscopy and hysteroscopy is also suggested in the literature and has been established as the gold standard for accurately diagnosing Müllerian anomalies (Fig. 2) [2].

**Fig. 3:**
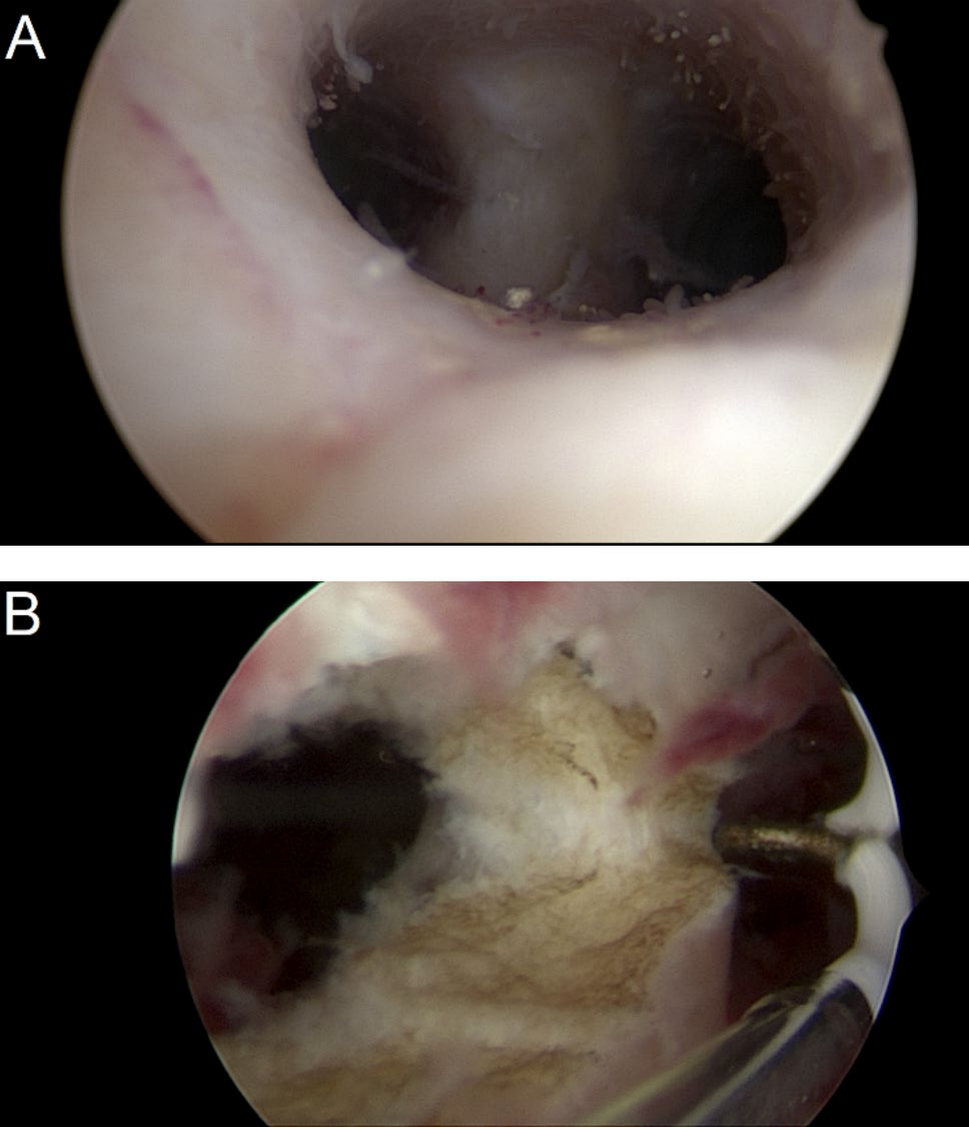
Types of uterus septum. **A** Subseptus, **B** Septus

Septal morphology was classified into three types (Fig. 3):

1. *Subseptus* the septum did not reach the cervical os.
2. *Septus* the septum extended to, but did not divide, the cervix.
3. *Septus completus* the septum involved both the uterine cavity and cervical canal.

For extensive septa (≥ 4–5 cm; septate or complete septate), we adopted a staged strategy, intentionally leaving a thin residual lamella at the index procedure to reduce the risk of uterine perforation and limit thermal spread. Completion resection was planned at second-look hysteroscopy. Intraoperative data collected included uterine perforation, hemorrhage, and fluid overload syndrome. In our center, patients receive standardized discharge instructions to return or contact us immediately during the first 4 postoperative weeks if any warning symptoms occur (fever, increasing pelvic pain, foul-smelling discharge, or heavy bleeding). Symptomatic patients are seen in our outpatient clinic by the surgical team. Postoperative complications—specifically endometritis—were recorded. Adhesion formation was assessed on routine second-look hysteroscopy, performed following complete septal resection. To prevent intrauterine adhesions, patients received either hyaluronic acid gel (Hyalobarrier®) alone or combined with a copper intrauterine device, selected according to septal size. Hyalobarrier® has been shown prospectively to reduce both the incidence and severity of adhesions after hysteroscopic surgery [15]. Second-look hysteroscopy was selectively scheduled for extensive septa (≥ 4–5 cm; septate or complete septate) and for planned two-stage resections. Routine second-look was not performed after limited subseptate resections without intraoperative concerns. These patients were followed clinically. Intrauterine adhesions were classified according to the European Society of Gynecological Endoscopy (ESGE) classification [16]. Furthermore, in cases of extensive dissection and absence of concomitant endometriosis, a three-month regimen of estrogen–progestin therapy (estradiol 4 mg daily on days 1–21 plus chlormadinone acetate 2 mg daily on days 9–21) was prescribed.

Data were analyzed to determine the safety profile of monopolar/bipolar energy modalities and to quantify adhesion risk and other perioperative and postoperative complications.

Prior to analysis, we performed a sample-size calculation based on a 2–3% prevalence in the general population to ensure adequate precision in estimating outcomes among women with a septate uterus. Assuming a 95% confidence level and a 5% margin of error, a prevalence of a septate uterus of 2% yields a required sample size of 31, while a prevalence of 3% requires 45 cases. With 155 patients (and 69 s look HSC) included in our study, we well exceed these minimums, providing sufficient power to assess safety outcomes, adhesion rates, and complication frequencies with confidence.

## Results

A total of 155 patients who underwent hysteroscopic septum dissection were included in this retrospective analysis. The mean age was 30.5 ± 5.3 years (range 17–44), and the mean body-mass index (BMI) was 24.7 ± 5.1 kg/m^2^ (range 16.5–40.1). Sterility was the indication for surgery in 30 patients (19.3%), while a history of abortion (total n = 59; 38.0%) was one of the most frequent clinical presentations. Given the well-documented association between Müllerian anomalies and endometriosis, we routinely evaluate patients in reproductive age presenting with endometriosis at our center for concurrent uterine malformations and their potential impact on fertility. When a septate uterus is identified, we offer simultaneous hysteroscopic septum dissection to optimize reproductive outcomes. Consequently, endometriosis was the primary indication in 63 (40.6%) of our 155 cases. Table 1 shows clinical indications.

**Table 1:** Clinical indications

Indication	Number of Patients (*n*)	Percentage (%)
Sterility	30	19.3
Abortus (total)	59	38
– 1 abortus	26	16.7
– 2 abortus	16	10.3
– 3 abortus	9	5.8
– > 3 abortus	8	5.1
Endometriosis	63	40.6
Preterm birth	2	1.3
Hematometra	1	0.6

Table 2 summarizes the distribution of septal types, septal length categories, and the energy modality employed for resection.

**Table 2:** Septum characteristics and treatment

Category	Number (*n*)	Percentage (%)
Septum type		
– Completus	19	12.2
– Septus	31	20.0
– Subseptus	105	67.7
Septum size		
– < 3 cm	52	33.5
– 3–5 cm	72	46.4
– > 5 cm	31	20.0
Type of Energy Used		
– Bipolar	101	65.1
– Monopolar	54	34.9

To minimize adhesion formation, 73 patients (47.1%) with subseptus received hyaluronic acid gel alone and 82 (52.9%) received hyaluronic acid gel plus a copper intrauterine device. Additionally, 10 patients with extensive septal resection and no concomitant endometriosis were prescribed postoperative estrogen–progestin therapy. In cases of extensive septa, we intentionally preserved a small septal remnant during the initial procedure to reduce the risk of uterine perforation and limit the extent of dissection; complete resection was then accomplished at second-look hysteroscopy. Second-look hysteroscopy was performed in 69/155 patients (44.5%), reflecting the subgroup with extensive or intentionally staged resections. Second-look hysteroscopy revealed intrauterine adhesions in three cases (2.8% grade I, 1.4% grade II), whereby one case (IUA I°) after bipolar resection and two cases occurred after monopolar resection (IUA I° and II°). Residual septal tissue was observed in 50 patients (72.5%) on second look, whereas 19 (27.5%) had a completely restored cavity. Although residual septal tissue was observed in 50/69 (72.5%), operative documentation did not consistently indicate whether the index resection was intentionally staged, and we therefore cannot disaggregate planned two-stage resections from conservative single-stage resections. Endometritis occurred in one patient after monopolar dissection (0.6%) (Table 3). No instances of uterine perforation, fluid overload syndrome, intraoperative hemodynamic instability, intra- or postoperative uterine bleeding, or post-ablation syndrome were observed in our cohort.

**Table 3:** Adhesions prophylaxis and outcomes

Outcome/Treatment	Number (*n*)	Percentage (%)
Hyaluron (for septum < 4 cm)	73	47.1
Hyaluron + IUD (for septum ≥ 4 cm)	82	52.9
Postoperative Estrogen + Gestagen (for patients with septus and septus completus 5 cm and without endometriosis)	10	6.4
Second-look Hysteroscopy (II° HSC)	69	44.5
IUA I°	2	2.8
IUA II°	1	1.4
Residual Septum	50	72.5
No Residual Septum	19	27.5
Endometritis	1	0.6

*IUA* Intrauterine adhesions, *IUD* Intrauterine device

Among patients with histologically confirmed endometriosis (n = 126, 81,3%), the prevalence was comparable across septum categories (Table 4).

**Table 4:** Endometriosis distribution by septum type

Septum type	Endometriosis (n/N)	Percentage (%)	*p* value
Completus	15 / 19	78.9	0.1031
Septus	25 / 31	80.6	
Subseptus	86 / 105	81.9	

*n* Number of patients with endometriosis, *N* total number of patients

*p* value was calculated using the chi-square test, with statistical significance defined as *p* < 0.05

These findings demonstrate a diverse range of septal morphologies and indications in our cohort, a predominance of bipolar energy use, low rates of adhesion formation and infectious complications, and a high coincidence of endometriosis across all septum types.

## Discussion

In this series of hysteroscopic septum resections using electrosurgical energy, we observed an excellent safety profile. Notably, there were no uterine perforations or significant hemorrhages, and only a single case of postoperative endometritis. This low adverse event rate is consistent with the general safety of operative hysteroscopy, where serious complications are uncommon. For example, a large prospective study found an overall operative hysteroscopy complication rate of about 0.95%, with uterine perforation in roughly 1.5% and significant bleeding in ~ 2.4% of cases [17]. Our low incidence of these complications (in total 4.8%, included IUA and endometritis) underscores that septal dissection with current techniques is very safe when performed by experienced surgeons. In our cohort, second-look hysteroscopy (performed in 44.5% of patients) revealed mild intrauterine adhesions in only 4.2%, confirming a very low adhesion rate. Severe intrauterine adhesions (III-IV°) were not observed. This is markedly lower than some previous reports using cold scissors; for instance, Hafizi et al. reported adhesions in 23% of cases after septoplasty with scissors [18]. Thus, electrosurgical dissection in our hands did not appear to increase adhesion formation and may be less injurious to the endometrial cavity.

Although this case series focused on surgical outcomes rather than long-term reproductive results, the literature is clear that removing a uterine septum enhances reproductive potential. Recent studies report substantial improvements in pregnancy and live birth rates after septum resection. For example, Davari Tanha et al. found that after hysteroscopic septoplasty in infertile women undergoing ART, the clinical pregnancy and live birth rates were 42.8% and 36.7%, respectively [19]. Meta-analyses similarly document that septum resection significantly reduces miscarriage rates. One systematic review concluded that treatment of a uterine septum “reduces the rate of spontaneous abortion” and ameliorates the adverse impact of the anomaly on pregnancy outcomes [20]. In sum, our findings of uncomplicated septum removal align with the broader evidence that electrosurgical metroplasty is an effective fertility-enhancing procedure.

Consistent with our safety data, the overall complication profile of hysteroscopic septum resection is very low. In our series only one patient required postoperative antibiotics (for endometritis) and no patient experienced post-ablation (post-resection) syndrome or significant fluid overload. In the literature, immediate complications of hysteroscopic surgery are rare: a large series reports hemorrhage in about 2.4% and perforation in about 1.5% of cases, while fluid overload syndromes occur in under 5% [17]. The absence of these problems in our cohort reflects both careful technique and modern equipment. Notably, we used bipolar resectoscopes in the majority of cases. Bipolar electrosurgery, which allows the use of isotonic saline as the distension medium, has been shown to virtually eliminate the hyponatremia and severe electrolyte shifts associated with monopolar (glycine) systems [21]. In practice, none of our patients developed hyponatremia or notable fluid imbalance. This is in line with guideline data showing that bipolar hysteroscopic resection maintains stable sodium levels even with modest fluid deficits [21].

When considering technique, it is worth comparing electrosurgical resection to purely mechanical approaches (cold scissors or micro scissors). Theoretically, cold scissors minimize thermal injury to adjacent endometrium, potentially preserving more normal tissue. In one recent trial, authors favored scissors for very thin septa and noted that patients treated with scissors had lower postoperative pain, but that both scissors and bipolar resectoscope achieved comparable septum removal and clinical outcomes [22]. Our study did not identify a clear disadvantage of electrosurgery; in fact, the near absence of adhesions in our series suggests that electrosurgical cutting—coupled with its instant coagulation—does not necessarily cause worse scarring than scissors. Electrosurgical resection may offer practical advantages: it allows controlled, progressive resection of fibrous septal tissue with continuous visualization and hemostasis. In contrast, scissors may require repeated instrument changes and can allow more bleeding during cutting, potentially obscuring the view. Both methods are valid, but current evidence indicates no major difference in reproductive outcome, and our experience shows that electrosurgical metroplasty is safe and effective.

In terms of efficiency, electrosurgical septum dissection is usually rapid and yields a clear field. The simultaneous cutting and coagulating action of a loop electrode means that intraoperative bleeding is minimal, providing excellent endoscopic visualization and expediency, contributing to the overall efficiency of hysteroscopic metroplasty with energy tools.

Finally, in clinical practice one must recognize that uterine septa often occur with other pathology. In our recent paper we highlighted that endometriosis is frequently found in women with Müllerian anomalies: in our cohort, nearly 77% of women with a septate uterus had histologically confirmed endometriosis [10]. This high coexistence means that when planning septum resection, clinicians should be vigilant for concomitant endometriosis, as treating both conditions may be necessary. In our experience, careful preoperative evaluation (often with imaging) and thorough inspection of the pelvis at the time of surgery are warranted so that any endometriosis can be managed alongside the septum.

In summary, our retrospective series demonstrates that hysteroscopic septum dissection using monopolar or bipolar electrosurgical energy is highly safe and effective. We observed no major complications, negligible adhesion formation, and a complication profile that compares favorably to historical data [17]. The approach appears to offer substantial fertility benefits, in line with published outcomes [19, 20]. Bipolar energy in particular permits safer fluid management, while still achieving excellent resection efficiency [21, 23]. When balanced against mechanical techniques, electrosurgical dissection provides rapid, precise cutting and hemostasis, yielding short operative times and good visualization. Given the frequent overlap with endometriosis, a comprehensive surgical plan remains important [10]. Overall, our findings support hysteroscopic electrosurgical metroplasty as a safe, effective, and efficient treatment for septate uterus in the gynecologic surgery setting.

## Strengths and limitations

This study benefits from a relatively large, well‐defined cohort of 155 consecutive cases—substantially exceeding the minimum sample size required to estimate outcomes with 95% confidence—thereby enhancing the precision of safety and adhesion‐rate estimates. Rigorous second‐look hysteroscopy in nearly half of the patients (44.5%) allowed direct assessment of residual septum and intrauterine adhesions, a feature not universally reported in prior series. Additionally, our routine use of diagnostic laparoscopy enabled simultaneous identification and management of endometriosis, reflecting real‐world multidisciplinary care. Finally, by including both monopolar and bipolar energy modalities under a unified “energy‐based” framework, the analysis captures contemporary practice and underscores the overall safety of electrosurgical metroplasty.

However, several limitations warrant consideration. The retrospective design carries inherent selection and information biases, and there was no randomized comparison against mechanical dissection or an untreated control group. Fertility outcomes and long‐term reproductive data were not systematically collected, limiting assessment of live birth rates. The frequency of intentionally staged resections was not systematically recorded in the operative notes, which precludes precise quantification. Adhesion rates derive from a selected second-look subgroup (extensive/staged cases) and may not generalize to all resections. As a single‐center study, findings may not generalize to all surgical settings or to operators with different levels of hysteroscopic experience.

## Conclusion

Hysteroscopic septum dissection using electrosurgical energy—whether monopolar or bipolar—is a safe and efficient procedure for correction of a septate uterus. In our series, no major perioperative complications occurred, adhesion formation was minimal, and postoperative infection was rare. Electrosurgical resection offers excellent visualization, rapid operative times, and effective hemostasis, while bipolar systems further reduce fluid‐management risks. Although prospective studies with long‐term fertility follow‐up are needed, our findings support energy‐based metroplasty as a low‐risk, fertility‐enhancing intervention in women with septate uteri.

## Data Availability

No datasets were generated or analyzed during the current study.
